# Development of a ^68^Ga‐labelled PET tracer for carbonic anhydrase IX‐overexpressed tumors using the artificial sweetener saccharin

**DOI:** 10.1002/jlcr.3893

**Published:** 2020-11-09

**Authors:** Un chul Shin, Jeong Su Choi, Yeon Jae Beak, Min woo Lee, Hyung Soo Kim, Dal Woong Choi, Dong Gil Kim, Suhng Wook Kim

**Affiliations:** ^1^ School of Health and Environmental Science, College of Health Science Korea University Seoul South Korea; ^2^ Health Science Research Center Korea University Seoul South Korea; ^3^ Kyung‐In Synthetic Corporation Seoul South Korea; ^4^ Transdisciplinary Major in Learning Health Systems Graduate School, Korea University Seoul South Korea

**Keywords:** ^68^Ga‐NOTA‐SAC, Carbonic anhydrase IX, PET tracer, Saccharin, U87MG

## Abstract

In this study, we developed a saccharin (SAC)‐based radiopharmaceutical (^68^Ga‐NOTA‐SAC) and evaluated the possibility of its application as a PET tracer in the diagnosis of carbonic anhydrase IX (CA IX)‐overexpressed tumors. We did a water‐soluble tetrazolium assay and flow cytometry analysis to identify the cell viability decrease by SAC. The radiochemical purity and stability of ^68^Ga‐ NOTA‐SAC in human and mouse serum was greater than 98%. The small animal PET image‐based radioactivity distribution of all organs decreased over time.^68^Ga‐NOTA‐SAC presented the highest tumor‐to‐muscle ratio at 90 min post injection (p.i). The growth rates of tumor‐to‐muscle ratios of ^68^Ga‐NOTA‐SAC were 88% at 60 min and 220% at 90 min, compared to 30 min p.i. The potential of ^68^Ga‐NOTA‐SAC as a PET tracer is expected to contribute to the diagnostic research on CA IX‐overexpressed tumors with the advantages of a relatively simple synthesis method.

## Graphical abstract

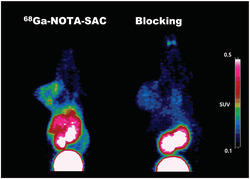

## 1 INTRODUCTION

Glycolytic metabolism is one of the most crucial processes of tumor cells. The anaerobic glycolytic pathway has been reported to cause acidic extracellular pH.^1^, ^2^ Carbonic anhydrase IX (CA IX), in an acidic tumor microenvironment, is known to be a representative isoenzyme that neutralizes the tumor acidification by acting as a catalyst in the mutual conversion between carbon dioxide and bicarbonate.^3^

The CA IX is a representative hypoxia‐induced enzyme in a broad range of solid tumors by hypoxia‐inducible‐factor‐1α (HIF‐1α) regulation.^4^ Overexpression of CA IX is mainly observed in malignant tumors, such as cervix, uteri, breast, lung, ovary, brain, and pancreas cancers.^5^, ^6^, ^7^, ^8^, ^9^ CA IX in a variety of solid tumors is a promising target for anti‐cancer therapeutics as well as an attractive ingredient (strategic site) for drug delivery, molecular imaging, and therapy.^10^, ^11^ Among the numerous inhibitors targeting CA IX, sulfonamides have been the most actively researched.^12^ Recently, a study reported that saccharin (SAC), an artificial sweetener, selectively targets CA IX as the zinc binding group.^13^ Furthermore, various SAC‐based sulfonamides were developed as inhibitors against CA IX.^14^ Also, SAC treatment (> 20mM) effectively inhibited the cell viability in an HT‐29 colon‐cell line.^15^ Therefore, a SAC‐based PET tracer could be used as a molecular imaging probe for the diagnosis of CA IX‐overexpressed tumors.

In this study, we developed a SAC‐based radiopharmaceutical as a probe for nuclear‐medicine imaging and evaluated the possibility of its application as a molecular image tracer in the diagnosis of CA IX‐overexpressed tumors. For this purpose, SAC was labeled using a ^68^Ga (*t*½ = 68 min; Eβ+, max = 1.92 MeV) radioisotope and bifunctional 1,4,7‐triazacyclononane‐1,4,7‐triacetic acid (NOTA). The synthesized ^68^Ga‐NOTA‐SAC was evaluated quantitatively by means of small‐animal micro PET with *in vitro/in vivo* assays in the pre‐clinical stage.

## 2 MATERIALS & METHODS

### 2.1 Cell viability assay

We purchased U87MG and fibroblast cells from the American Type Culture Collection (ATCC, Manassas, VA, USA). We did a cell‐viability assay using a Quanti‐MAX™ WST‐8 Cell Viability Assay Kit (Biomax, Seoul, Korea). Briefly, the cells were seeded in 96‐well plates at a density of 2 x 10^4^ cells/well and incubated at 37°C in a humidified 5.0% CO_2_ atmosphere under normoxic and hypoxic conditions. We used the BD GasPak EZ Pouch system (Becton, Dickinson and Company, Franklin Lakes, NJ, USA) to induce hypoxic condition.^16^ The cultured cells were treated with sodium saccharin or NOTA‐SAC conjugates at various concentrations (twofold serial dilutions ranging from 50 to 0.098mM) in each well for 24 h. After treatment, we added WST‐8 reagent (20 μL) to each well, incubated them for 1 h, and measured the absorbance at 450 nm using a microplate reader (SpectraMax 190, Molecular Devices, Downingtown, PA, USA) to confirm cell viability. U87MG cells, cultured at a density of 3 x 10^5^ cells/well in 6‐well plates under normoxic and hypoxic condition for 24 h, were treated with sodium saccharin (12.5mM) to re‐evaluate the cytotoxicity by FACS analysis using an Annexin V‐FITC Apoptosis detection kit (Invitrogen, CA, USA). We did the FACS analysis using a flow cytometer (FACS Lyric BD; BD Biosciences, Franklin Lakes, NJ, USA).

### 2.2 Preparation and HPLC analysis of NOTA‐SAC conjugates

We purchased the NOTA from Macrocyclic, Inc. (Dallas, TX) and the rest of the chemicals from Sigma‐Aldrich Chemical (St. Louis, MO). SAC was provided by JMC (Jeil Moolsan Company, Ulsan, Korea). A solution of sodium saccharin (1.83 mg, 8.9 μmol) in distilled water (200 μL) was added to a sodium bicarbonate solution (1 N, 200 μL, pH 8.5) including NOTA (1 mg, 1.8 μmol). The mixture was stirred at room temperature for 24 h. The NOTA‐SAC conjugates were filtered by means of a size‐exclusion column (PD10 column) with base‐solution change using aqueous sodium acetate (pH 5.5, 1mM).

We did HPLC (high performance liquid chromatography) analyses on a HITACH chromaster (Hitachi, Japan) consisting of a 5,110 Pump, 5,280 autosampler, 5,310 Column oven (40°C), and 5,410 UV detector. We did chromatographic separation on a TSKgel ODS‐100 V 5 um (4.6 mm I. D x 15 cm) column using a mobile phase consisting of sodium phosphate buffer‐methanol (50:50 v/v) at a flow rate of 1 ml/min with a UV detection wavelength of 270 nm with 2 μL injection volume.

### 2.3 Tandem mass spectrometry analysis

We used a Tandem mass spectrometer system (Quantiva, Thermo Scientific, USA) for identification of the conjugated substance. We applied an electrospray ionization (ESI) mode and analyzed the NOTA and SAC in negative mode. The mass range was basically set to 100 to 1700 m/z in order to confirm the ratio of the combination of NOTA and sodium saccharin. Also, for detailed identification, the mass range was subdivided (100‐400, 400‐800, 800‐1,200, and 1,200‐1700 m/z) and confirmed. Capillary voltage was set to 3,500 V, sheath gas was set to 35 Arb, and aux gas was set to 2 Arb. First, we used multiple reaction mode (MRM) analysis to identify fragment ions of individual substances of NOTA and SAC. The standard stock solution of each substance was diluted with ultrapure water (18.2 MΩ cm) to confirm the Q1 value, and then applied the CE value to identify the fragment ions (Q3). The prepared sample was also diluted 10 times with ultrapure water, and then analyzed. We prepared ultrapure water for tandem mass spectrometry analysis by using an aquaMAX™ Ultra 370 series (YL Instruments, Korea) water purification system.

### 2.4 Synthesis of ^68^Ga‐labeling NOTA‐SAC conjugates and Radio‐TLC analysis

The ^68^GaCl_3_ was eluted with 5 ml of 0.1 M hydrochloric acid from a ^68^Ge/^68^Ga generator (ITG, U.S.A). We measured the radioactivity using a calibrated‐dose calibrator (Capintec CRC‐15R, U.S.A). ^68^GaCl_3_ (370 MBq) was dried by means of purging with nitrogen (99.9999%) at 100°C and was later added to a solution of NOTA‐SAC conjugate in sodium acetate buffer (0.5 mL, 1mM, pH 5.5). The reaction mixture was incubated for 10 min at 80°C. After labeling ^68^Ga, we used the product without any further purification. The radiochemical purity was analyzed by means of ITLC (mobile phase of citrate buffer, pH 5 and 0.1 M) using an AR‐2000 radio TLC scanner (Eckert & Ziegler, U.S.A).

### 2.5 Octanol‐water partition coefficient (Log p value) and serum stability test

The octanol/water partition coefficient of ^68^Ga‐NOTA‐SAC was found based on the following protocol. A solution of ^68^Ga‐labeled conjugate was added (3.7 MBq) to octanol (0.5 mL) in PBS (0.5 mL, pH 7.4); the mixture was stirred vigorously for at least 10 min and then centrifuged at 12,500 rpm for 5 min. We collected aliquots of the aqueous and octanol phases (200 μL each) and calculated the log *p* values (*n* = 3) in the gamma counter (1,480 Wizard3, Perkin Elmer). For the serum stability test, ^68^Ga‐NOTA‐SAC conjugates (18.5 MBq) mixed with either human or mouse serum (0.5 mL) were incubated at 37°C for different time intervals (1 h, 3 h, and 6 h).

### 2.6 Cell culture and tumor xenografts

U87MG cultured in DMEM (Dulbecco's Modified Eagle Medium, Thermo Fisher, NY, USA) augmented with 10% fetal bovine serum and 1% penicillin‐streptomycin in 5% CO_2_ at 37°C. BALB/c nu/nu female mice (age, six weeks) weighing 20 g were purchased from NARA Biotec, Inc (Seoul, Korea). We subcutaneously implanted U87MG (1 × 10^6^ cells in 100 μL DMEM) in the left shoulder region of each mouse. The mice were then subjected to bio‐distribution and PET imaging after the tumor volume reached 1.0 ~ 1.5 cm^3^ in diameter (14 ~ 18 days after xenografts). All animal procedures conducted were in line with the protocol approved by the animal research committee of Korea University (KUIACUC‐2019‐0010).

### 2.7 Biodistribution studies

Tumor‐bearing mice were anesthetized with 1.5% isoflurane (Abbott Lab., Ltd, USA) mixed with 35% O_2_ in N_2_. ^68^Ga‐NOTA‐SAC was injected with 370 kBq by means of the tail vein of each mouse (*n* = 4). The mice were sacrificed by neck drawing at different intervals (30, 60, and 90 min) p.1. Organs of interest (such as muscles, liver, kidneys, bone, lung, spleen, heart, intestine, stomach, tail, and blood) and tumors were harvested and measured for radioactivity using a gamma counter (1,480 Wizard3, Perkin Elmer). We calculated the mean uptake for an organ of interest by means of standard deviations and expressed it as a percentage of the injected dose per gram (%ID/g).

### 2.8 Micro PET image acquisition

We acquired small‐animal PET images by means of a micro PET scanner (Inveon PET, Siemens, Knoxville, TN, USA). Approximately 74 MBq (200 μCi/100 μL) of free ^68^Ga, ^68^Ga‐NOTA, and ^68^Ga‐NOTA‐SAC were intravenously administrated via the tail vein of each mouse (*n* = 4) under 1.5% isoflurane anesthesia. We did the CA IX blocking study by I. V injection of unradiolabelled‐pure saccharin (10 mg) first and then I. V injection of ^68^Ga‐NOTA‐SAC (74 MBq/100 μL) after about 1 h.

We acquired dynamic PET images at 90 min post injection (p.i) as whole‐body imaging. The acquired list mode PET images were reconstructed as three static frames (each 30 min) using a two‐dimensional order‐subset expectation maximization (OSEM)3D/SP‐MAP algorithm (OESM3D, 2 iterations; SP‐MAP, 18 iterations). The reconstructed dynamic PET images were then converted to standard uptake value (SUV), and the regions of interest (ROIs) were defined in the tumor region, liver region, and kidney region based on the whole‐body images. We quantitatively evaluated the distribution of radioactivity in each ROI by means of Inveon Research Workplace software (Siemens, Knoxville, TN, USA).

### 2.9 Statistical analysis

The data of SUVs and tumor to muscle ratio based on PET image were analyzed by independent t‐test using the SPSS software package (SPSS, Version 12.0, Chicago, IL, USA). All data in the figures are expressed as the mean and standard deviation of three independent experiments and statistical significance was accepted for p‐values less than 0.05.

## 3 RESULTS

### 3.1 Cell‐viability test

**FIGURE 1:**
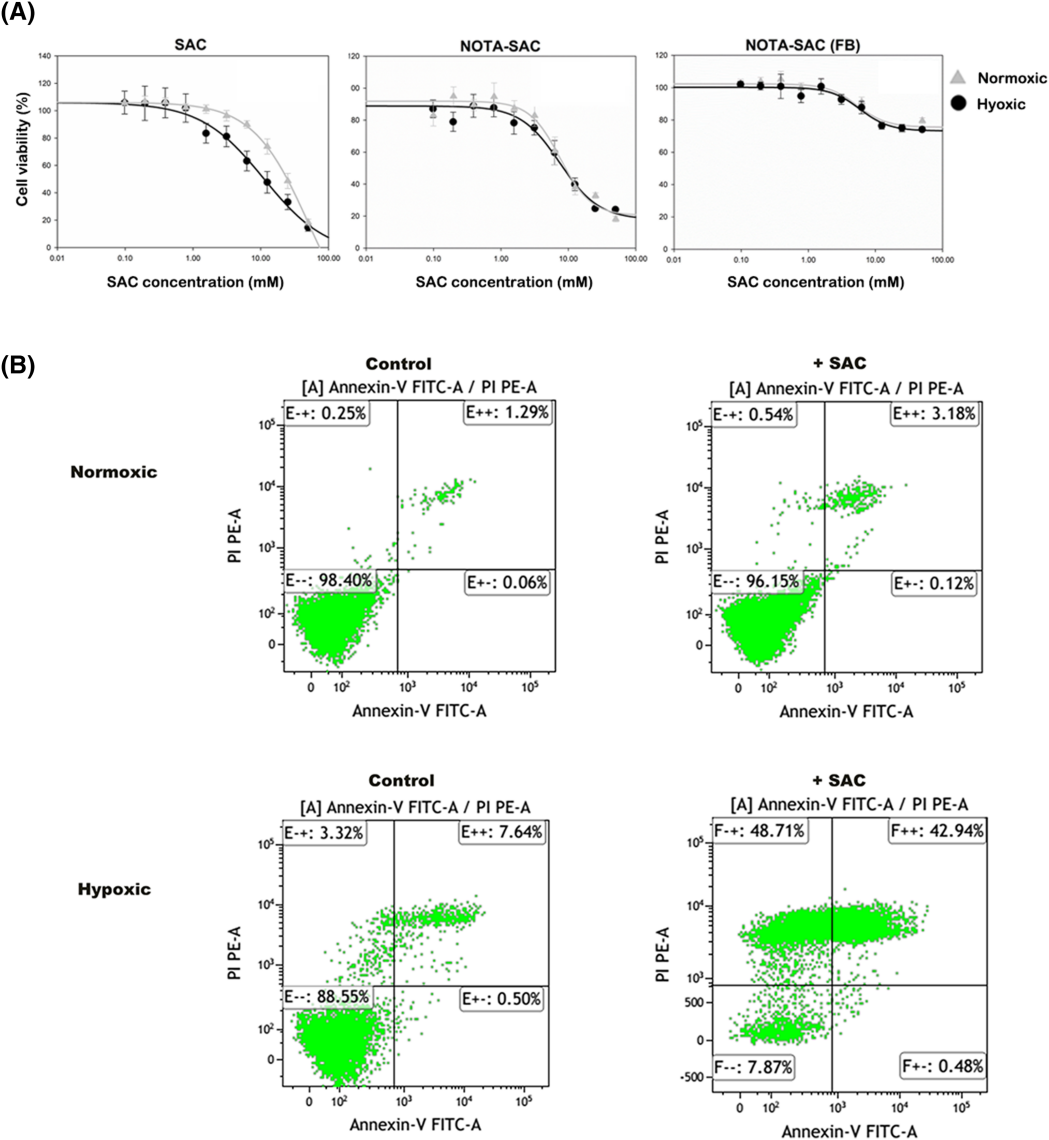
Cell viability test of SAC or NOTA‐SAC conjugates treated U87MG and fibroblast cell in normoxic and hypoxic conditions. A, Evaluation results of U87MG and fibroblast cell viability by treatment with SAC or NOTA‐SAC conjugates in normoxic and hypoxic conditions, and B, Distribution of apoptotic and necrotic cell populations in SAC‐untreated (control)/treated (+SAC) U87MG cells using flow cytometry

We tested the viability of the CA‐IX‐expressing U87MG human glioma tumor‐cell line, because the saccharin selectively targets CA‐IX. First, we tested the cytotoxicity of saccharin against U87MG cells in normoxic and hypoxic conditions is shown in Figure 1A. In the normoxic and hypoxic conditions, the viability of U87MG decreased in a concentration‐dependent manner. However, we confirmed that the sensitivity of U87MG to saccharin was higher in the hypoxic condition (IC_50_ value, 9.9 ± 1.7mM) than in the normoxic condition (IC_50_ value, 22.0 ± 2.1mM). The NOTA‐SAC conjugates also showed similar result as saccharin in U87MG cell viability assay (IC50 value in normoxic condition, 11.1 ± 2.8 and IC50 value in hypoxic condition, 20.8 ± 2.9), suggesting that the specificity of NOTA‐SAC conjugate to CA IX is similar to specificity of saccharin. Moreover, the NOTA‐SAC conjugates showed low cytotoxicity under the both normoxic and hypoxic conditions in the fibroblast cell as a normal control, unlike the U87MG which CA IX is overexpressed. The results of viable, apoptotic, and necrotic cell‐population analysis using FACS are shown in Figure 1B. The statistically significant decrease in the population of viable cells was also observed, from 78.21 ± 13.95% to 7.91 ± 3.80%, owing to SAC treatment in the hypoxic condition (*p* = 0.019).

### 3.2 Evaluation of NOTA‐SAC conjugates

**FIGURE 2:**
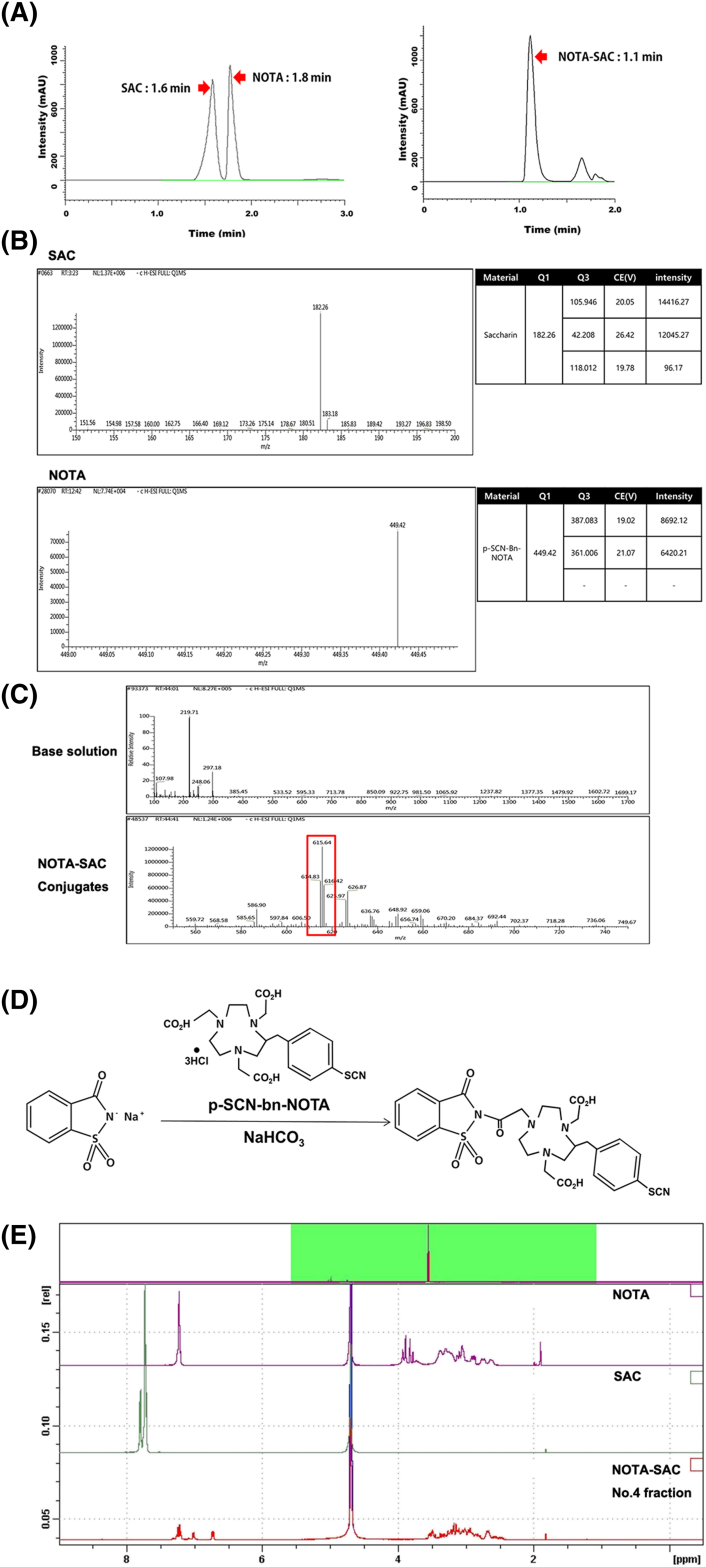
Evaluation of NOTA‐SAC conjugates using HPLC and mass spectroscopy. A, Comparison of detection time of NOTA, SAC, and NOTA‐SAC conjugates (arrows) by HPLC analysis. B, Control m/z values of optimized NOTA and SAC. C, m/z values of the base solution and NOTA‐SAC conjugates (square box). D, Synthesis process and possible structure of the NOTA‐SAC conjugate. E, Results of 1H‐NMR analysis of SAC, NOTA and NOTA‐SAC conjugates

SAC was conjugated with NOTA for ^68^Ga‐labeling, and NOTA‐SAC conjugates were evaluated using HPLC and tandem mass spectrometry. In the HPLC analysis, NOTA and SAC were detected at 1.8 and 1.6 min, respectively, and NOTA‐SAC conjugates were detected separately from NOTA and SAC at 1.1 min (Figure 2A). Individual mass values of NOTA and SAC were detected as 182 and 449 m/z by tandem mass spectrometry, respectively (Figure 2B). The mass value of NOTA‐SAC conjugates was detected as 615 m/z by tandem mass spectrometry, indicating that one of the three hydroxyl groups of NOTA was replaced by saccharin (Figure 2C). Furthermore, only a mass value corresponding to 1:1 was identified, showing that NOTA and SAC were conjugated in a ratio of 1:1The possible structure of the NOTA‐SAC conjugate is shown in Figure 2D. This structure was also confirmed by ^1^H‐NMR experiments (Figure 2E).

### 3.3 Synthesis and in vitro Characterization of ^68^Ga‐NOTA‐SAC

**FIGURE 3:**
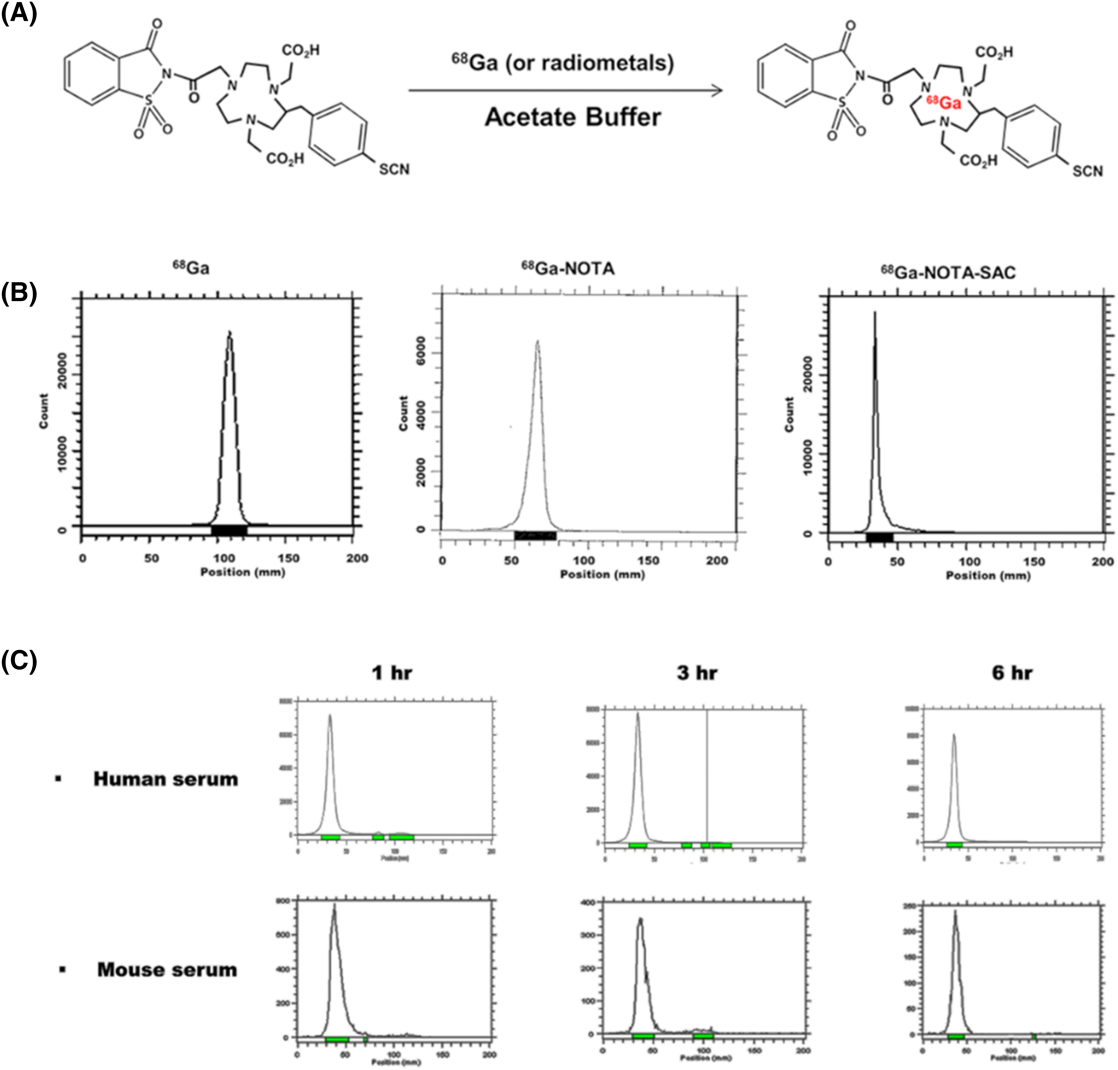
Radiolabeling process of ^68^Ga‐labelled NOTA‐SAC and radio‐TLC results. A, Process of ^68^Ga chelating to NOTA‐SAC conjugates and synthesized ^68^Ga‐NOTA‐SAC structure, B, Results of radio‐TLC analysis of ^68^Ga, ^68^Ga‐NOTA, and ^68^Ga‐NOTA‐SAC, C, Results of serum stability in human and mouse serum, respectively

The ^68^Ga‐labeling technique with SAC was optimized in sodium acetate buffer (pH 5.5) to attain high labeling efficiency (Figure 3A). The radiochemical purity of the ^68^Ga‐NOTA‐SAC was greater than 98% (Figure 3B). The ^68^Ga‐NOTA‐SAC in human and mouse serum was highly stable radiochemically (> 98%) using radio‐TLC analysis for over 6 h (Figure 3C). We assessed the hydrophilicity of the ^68^Ga‐NOTA‐SAC via the octanol‐water partition coefficient measurements. The measured log *p* value was ‐1.12 ± 0.22.

### 3.4 Biodistribution of ^68^Ga‐NOTA‐SAC

**FIGURE 4:**
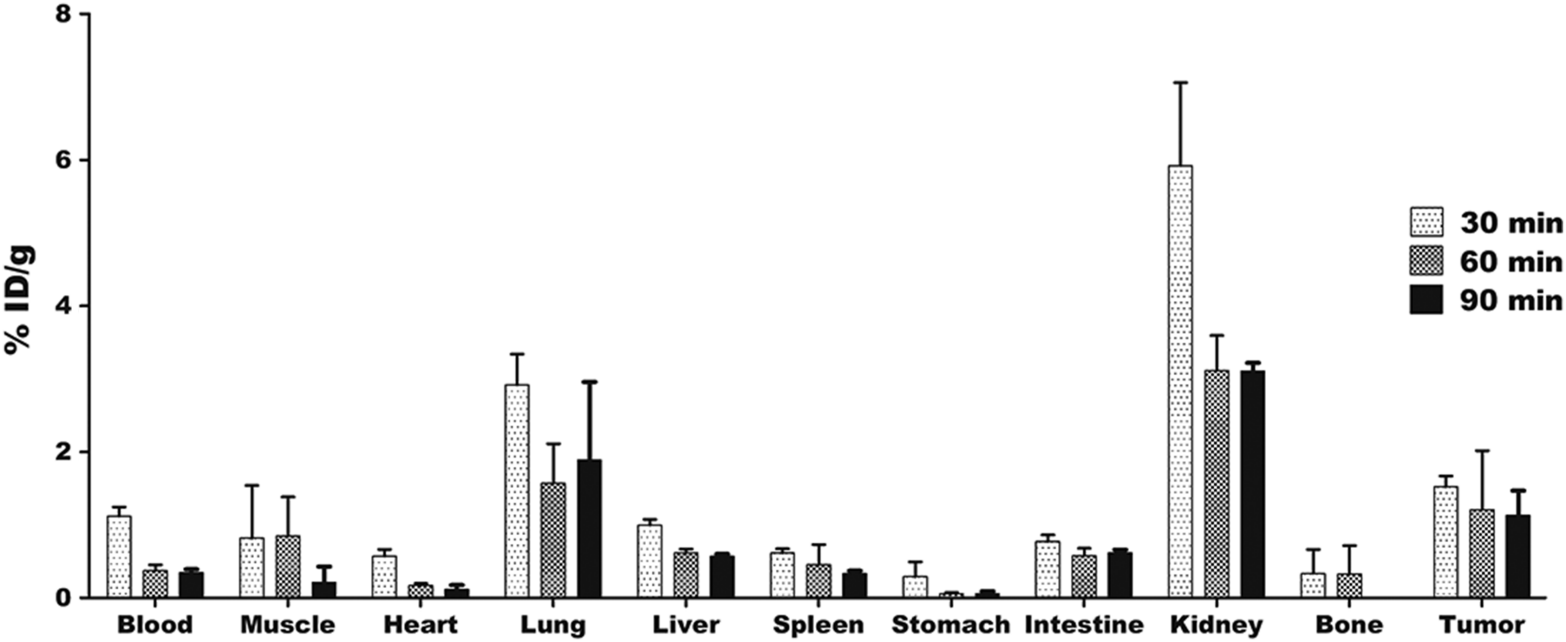
Biodistribution of ^68^Ga‐NOTA‐SAC in U87MG xenografted mouse models. Uptake patterns of the radioactivity distribution in each organ at 30, 60, and 90 min

The ^68^Ga‐NOTA‐SAC biodistribution was reduced over time in most of the organs except for the lung region (Figure 4). After 30 min, the ^68^Ga‐NOTA‐SAC in the blood‐pool rapidly decreased as to 1.1% ID/g (30 min p.i), 0.3% ID/g (60 min p.i), and 0.3%ID/g (90 min p.i). Tumor uptake was 1.5% ID/g (30 min p.i), 1.2% ID/g (60 min p.i), and 1.1% ID/g (90 min p.i). The distribution in the muscle region showed a rapid decrease at 90 min p.i, and kidneys were evaluated as decreasing rapidly after 30 min p.i as the organ with the highest distribution.

### 3.5 PET images of free ^68^Ga, ^68^Ga‐NOTA, and ^68^Ga‐NOTA‐SAC

**FIGURE 5:**
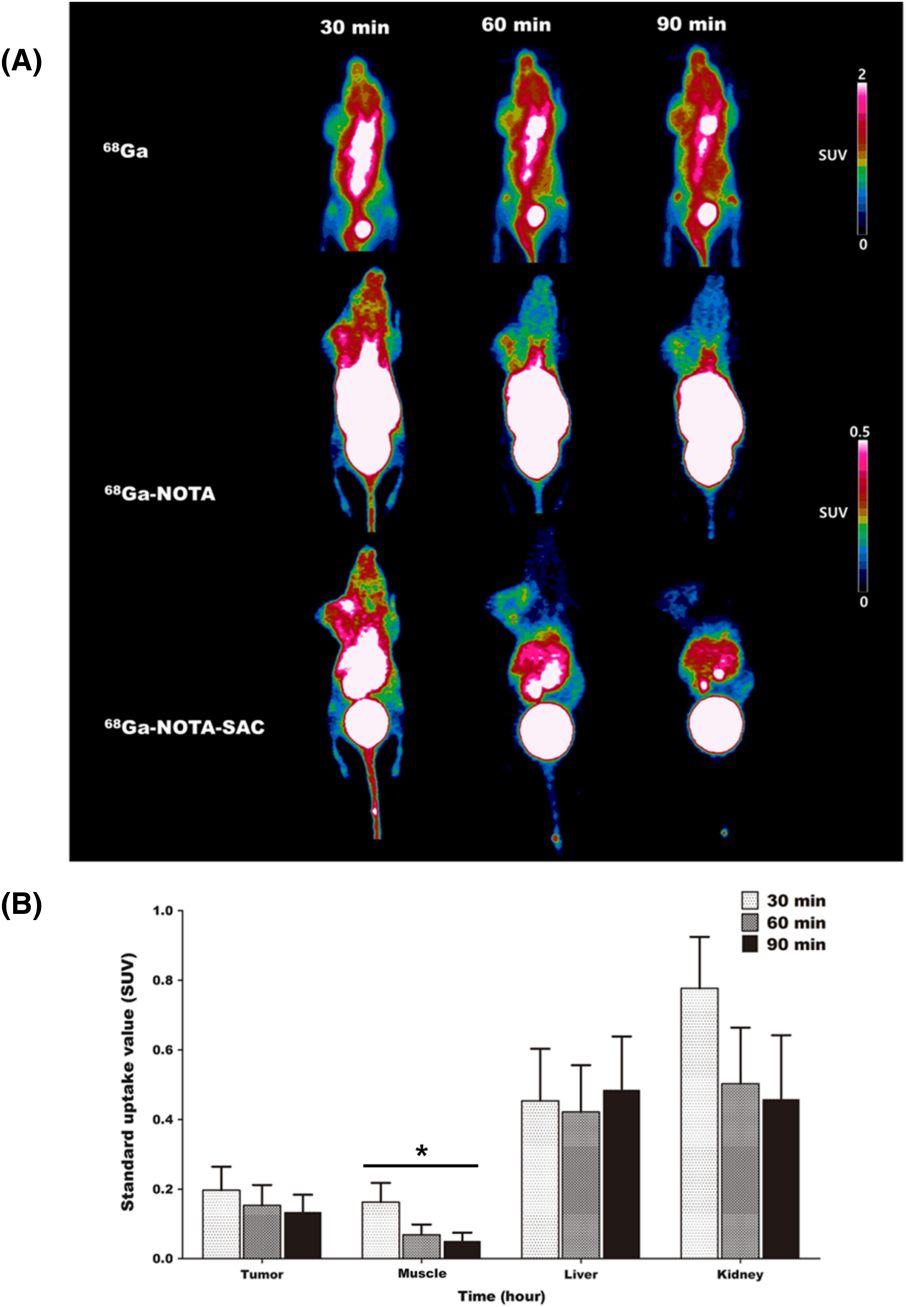
PET image analysis of free ^68^Ga, ^68^Ga‐NOTA, and ^68^Ga‐NOTA‐SAC. A, Comparison of PET images at 30, 60, and 90 min, respectively. B, Analysis of time‐dependent SUV values in ^68^Ga‐NOTA‐SAC injected mouse models

`Small‐animal PET images, reconstructed using three frames, demonstrated the *in vivo* distribution of the free ^68^Ga, ^68^Ga‐NOTA and ^68^Ga‐NOTA‐SAC in the whole body over time (Figure 5A). Free ^68^Ga, ^68^Ga‐NOTA, and ^68^Ga‐NOTA‐SAC exhibited the most intensive distribution of radioactivity at 30 min p.i in the kidney and liver regions (Table 1). The radioactivity distribution of each organ decreased as a whole over time. Free ^68^Ga showed a somewhat higher distribution of radioactivity in the liver region than in the kidney region in the entire time domain. ^68^Ga‐NOTA and ^68^Ga‐NOTA‐SAC, in contrast, presented a higher distribution of radioactivity in the kidney region than in the liver region. ^68^Ga‐NOTA‐SAC exhibited the lowest radioactivity distribution in the liver and kidney region over the entire time domain compared to ^68^Ga and ^68^Ga‐NOTA (Figure 5B).

**TABLE 1:** Comparison of PET image‐based tumor‐to‐muscle ratio of ^68^Ga, ^68^Ga‐NOTA, and ^68^Ga‐NOTA‐SAC

Model	Tumor/muscle ratio		
	30 min	60 min	90 min
^**68**^ **Ga**	**1.25 ± 0.02**	**1.31 ± 0.03**	**1.39 ± 0.02**
^**68**^ **Ga‐NOTA**	**1.98 ± 0.21**	**2.18 ± 0.32**	**2.13 ± 0.24**
^**68**^ **Ga‐NOTA‐SAC**	**1.3 ± 0.09**	**2.45 ± 0.14**	**4.17 ± 1.04**

### 3.6 Blocking

**FIGURE 6:**
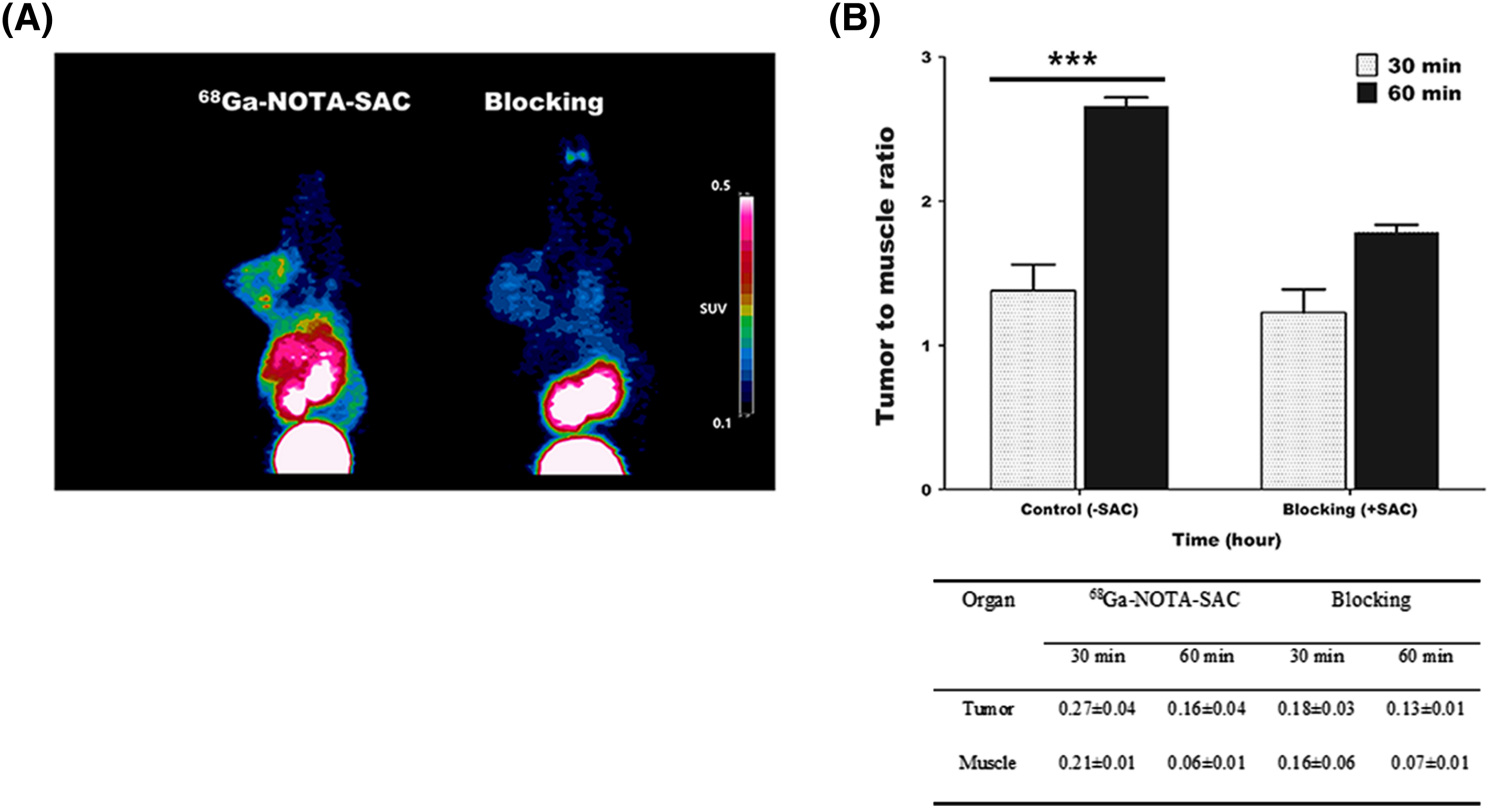
SAC‐blocking PET image analysis in U87MG xenografted mouse models. A, Comparison of acquired PET images at 60 min. B, Tumor‐to‐background ratio and SUVs of ^68^Ga‐NOTA‐SAC in the control model (‐SAC) and blocking model (+SAC)

To identify the specificity of ^68^Ga‐NOTA‐SAC to U87MG tumors, we did blocking experiments using pure SAC. When pure SAC was pre‐treated to block the binding of ^68^Ga‐NOTA‐SAC to U87MG tumors, the expected blocking PET images were acquired (Figure 6A). In blocking PET images, the tumor‐to‐muscle ratio of ^68^Ga‐NOTA‐SAC did not show a significant difference in radioactivity distribution at the tumor site compared to the muscle. The tumor‐to‐muscle ratios were 1.22 ± 0.16 at 30 min and 1.78 ± 0.05 at 60 min (Figure 6B).

## 4 DISCUSSION

Tumor acidification is known to be caused by the anaerobic glycolysis in tumor cells. Despite sufficient oxygen supply in the tumor area, tumor acidification progresses by means of anaerobic glycolysis.^17^, ^18^ The unique metabolism of tumor cells produces large amounts of lactic acid and hydrogen ions, and the acidic microenvironment affects the metastasis and malignancy of these tumors.^19^ CA IX, as a zinc metalloenzyme, is one of the transmembrane CA isoenzymes overexpressed to neutralize the pH of acidified tumor cells.^20^ The CA IX is widely used as a tumor‐associated marker for diagnosis and selective therapy in cancer cells.^21^

Recently, sulfonamides have been actively researched as a potential CA IX tracer that could selectively bind CA IX over‐expressed tumors.^22^ Saccharin, one of the sulfonamides, has a sweetness that is about 400 times higher than that of sucrose and is generally used as a calorie‐free sugar substitute in food products as artificial sweeteners.^23^ Saccharin is moderately absorbed in the human organs without metabolism as a sulfonamide and is rapidly excreted from the blood pool by the kidneys.^24^

Based on this concept, we developed a saccharin‐based PET radiopharmaceutical for the diagnosis of CA IX‐overexpressed tumor cells and quantitatively evaluated the properties of ^68^Ga‐NOTA‐SAC by an *in vitro* and *in vivo* study.

Firstly, we induced CA IX in U87MG cells using hypoxic pouch system,^16^ and performed a cell‐viability assay and flow cytometry study for the evaluation of SAC based CA IX tracer ability. Through results of these studies, we confirmed the potential of SAC as a CA IX tracer by determination of anti‐proliferative effect of SAC on CA IX expressed U87MG cells.

Next, we developed a synthetic method for NOTA‐SAC conjugation and were able to synthesize ^68^Ga‐labelled a NOTA‐SAC PET tracer quickly and stably. This synthesis method could cover the characteristics of the gallium, which has a relatively short half‐life. In the biodistribution study of ^68^Ga‐NOTA‐SAC, radioactivity decreased over time in most organs, and this reduction rate was even more increased 30 min p.i. The tumor region was also rapidly decreased after 30 minutes p.i., but the reduction rate was relatively low over time compared to that of other organs. This rapid excretion of ^68^Ga‐NOTA‐SAC in internal organs might be influenced by a characteristic of saccharin, which has been confirmed in research associated with saccharin excretion.^25^

These distribution characteristics of ^68^Ga‐NOTA‐SAC were also demonstrated by means of PET images of U87MG xenografted mice. Despite the rapid decrease in the tumor region after 30 min p.i, the tumor‐to‐muscle ratio was increased over time than in other organs. The rapid excretion of ^68^Ga‐NOTA‐SAC is an important property that not only improves the PET images contrast but also reduces various biological side effects caused by exposure in the diagnostic field using radioactive isotopes.

We compared the ability of the CA IX tracer of ^68^Ga‐NOTA‐SAC with free ^68^Ga and ^68^Ga‐NOTA using PET images. Free ^68^Ga did not demonstrate selective PET tumor images, because the tumor‐to‐muscle ratio of free ^68^Ga presented the highest value as 1.41 ± 0.02. Although the tumor‐to‐muscle ratio of ^68^Ga‐NOTA was higher than that of free ^68^Ga, the highest value of ^68^Ga‐NOTA was only 2.18 ± 0.32. However, ^68^Ga‐NOTA‐SAC demonstrated better contrast in selective PET tumor images than did ^68^Ga‐NOTA that was not conjugated with SAC. ^68^Ga‐NOTA‐SAC presented the highest tumor‐to‐muscle ratio as 4.17 ± 1.04. In addition, the growth rates of the tumor‐to‐muscle ratios of ^68^Ga‐NOTA‐SAC were increased from 88% at 60 min to 220% at 90 min compared to 30 min p.i. In addition, although saccharin showed a moderate blocking effect at 30 minutes due to the rapid excretion rate in the body, the CA IX traceability of saccharin have been limitedly evaluated through a blocking study.

## 5 CONCLUSIONS

In conclusion, we developed a new ^68^Ga‐NOTA‐SAC as a saccharin‐based PET tracer for diagnosis of CA IX‐overexpressed tumors and quantitatively assessed the targeting ability of ^68^Ga‐NOTA‐SAC using micro‐PET imaging with *in vitro* experiments. These ^68^Ga‐NOTA‐SACs demonstrated a high uptake ratio of the tumor region as well as rapid excretion, allowing a high‐contrast PET image of the targeted tumor region. The SAC has advantages, such as relatively high *in vivo* stability with economic efficiency, and ^68^Ga has spatial and temporal advantages, because it was produced by means of a generator. The new ^68^Ga‐NOTA‐SAC is expected to be applicable to the diagnosis of CA IX‐overexpressed tumors with the advantage of a relatively simple synthesis method.

## AUTHOR CONTRIBUTIONS

Dong Gil Kim and Suhng Wook Kim designed, planned and oversaw the project. Un chul Shin and Jeong Su Choi performed the experiments and analyzed the results. Un chul Shin, Yeon Jae Beak and Min woo Lee wrote the manuscript. Dong Gil Kim and Suhng Wook Kim interpreted the results and gave feedback on the project to the senior authors, Hyung Soo Kim and Dal Woong Choi built experimental equipments and setups. All authors provided editorial comments and approved the final version of the manuscript.

## DISCLOSURE STATEMENT

The authors disclose no conflict of interest.

## Data Availability

The datasets used and/or analyzed during the current study are available from the corresponding author on reasonable request.

## References

[1] Kato Y , Ozawa S , Miyamoto C , et al. Acidic extracellular microenvironment and cancer. Cancer Cell Int. 2013;13(1):1‐8. 24004445 10.1186/1475-2867-13-89 PMC3849184

[2] Paradise RK , Lauffenburger DA , Van Vliet KJ . Acidic extracellular pH promotes activation of integrin α_v_β_3_ . PLoS ONE. 2011;6(1):e15746. 10.1371/journal.pone.0015746 21283814 PMC3023767

[3] Švastová E , Hulíková A , Rafajová M , et al. Hypoxia activates the capacity of tumor‐associated carbonic anhydrase IX to acidify extracellular pH. FEBS Lett. 2004;577(3):439‐445. 15556624 10.1016/j.febslet.2004.10.043

[4] Lendahl U , Lee KL , Yang H , Poellinger L . Generating specificity and diversity in the transcriptional response to hypoxia. Nat Rev Genet. 2009;10(12):821‐832. 19884889 10.1038/nrg2665

[5] Chia SK , Wykoff CC , Watson PH , et al. Prognostic significance of a novel hypoxia‐egulated marker, carbonic anhydrase IX, in invasive breast carcinoma. J Clin Oncol. 2001;19(16):3660‐3668. 11504747 10.1200/JCO.2001.19.16.3660

[6] Lou Y , McDonald PC , Oloumi A , et al. Targeting tumor hypoxia: suppression of breast tumor growth and metastasis by novel carbonic anhydrase IX inhibitors. Cancer Res. 2011;71(9):3364‐3376. 21415165 10.1158/0008-5472.CAN-10-4261

[7] Ilie M , Mazure NM , Hofman V , et al. High levels of carbonic anhydrase IX in tumour tissue and plasma are biomarkers of poor prognostic in patients with non‐small cell lung cancer. Br J Cancer. 2010;102(11):1627‐1635. 20461082 10.1038/sj.bjc.6605690 PMC2883156

[8] Järvelä S , Parkkila S , Bragge H , et al. Carbonic anhydrase IX in oligodendroglial brain tumors. BMC Cancer. 2008;8(1):1‐9. 18173856 10.1186/1471-2407-8-1 PMC2245965

[9] Ivanov S , Liao SY , Ivanova A , et al. Expression of hypoxia‐inducible cell‐surface transmembrane carbonic anhydrases in human cancer. Am J Pathol. 2001;158(3):905‐919. 11238039 10.1016/S0002-9440(10)64038-2 PMC1850340

[10] Supuran CT . Inhibition of carbonic anhydrase IX as a novel anticancer mechanism. J Clin Oncol. 2012;3(7):98‐103. 10.5306/wjco.v3.i7.98 PMC3394083 22787577

[11] Stillebroer AB , Mulders PF , Boerman OC , Oyen WJ , Oosterwijk E . Carbonic anhydrase IX in renal cell carcinoma: implications for prognosis, diagnosis, and therapy. Eur Urol. 2010;58(1):75‐83. 20359812 10.1016/j.eururo.2010.03.015

[12] Monti SM , Supuran CT , De Simone G . Carbonic anhydrase IX as a target for designing novel anticancer drugs. Curr Med Chem. 2012;19(6):821‐830. 22214452 10.2174/092986712799034851

[13] Ivanova J , Carta F , Vullo D , et al. N‐Substituted and ring opened saccharin derivatives selectively inhibit transmembrane, tumor‐associated carbonic anhydrases IX and XII. Bioorg Med Chem. 2017;25(13):3583‐3589. 28416101 10.1016/j.bmc.2017.04.007

[14] Mahon BP , Hendon AM , Driscoll JM , et al. Saccharin: a lead compound for structure‐based drug design of carbonic anhydrase IX inhibitors. Bioorg Med Chem. 2015;2(4):849‐854. 10.1016/j.bmc.2014.12.030 PMC4352949 25614109

[15] van Eyk AD . The effect of five artificial sweeteners on Caco‐2, HT‐29 and HEK‐293 cells. Drug Chem Toxicol. 2015;38(3):318‐327. 25317478 10.3109/01480545.2014.966381

[16] Jhaveri N , Cho H , Torres S , et al. Noscapine inhibits tumor growth in TMZ‐resistant gliomas. Cancer Lett. 2011;312(2):245‐252. 21925789 10.1016/j.canlet.2011.08.015

[17] Payen VL , Porporato PE , Baselet B , Sonveaux P . Metabolic changes associated with tumor metastasis, part 1: tumor pH, glycolysis and the pentose phosphate pathway. Cell Mol Life Sci. 2016;73(7):1333‐1348. 26626411 10.1007/s00018-015-2098-5 PMC11108399

[18] Sandoz G , Douguet D , Chatelain F , Lazdunski M , Lesage F . Extracellular acidification exerts opposite actions on TREK1 and TREK2 potassium channels via a single conserved histidine residue. Proc Natl Acad Sci. 2019;106(34):14628‐14633. 10.1073/pnas.0906267106 PMC2732798 19667202

[19] Justus CR , Dong L , Yang LV . Acidic tumor microenvironment and pH‐sensing G protein‐coupled receptors. Front Physiol. 2013;4:354. 10.3389/fphys.2013.00354 24367336 PMC3851830

[20] Wingo T , Tu C , Laipis PJ , Silverman DN . The catalytic properties of human carbonic anhydrase IX. Biochem Biophys Res Commun. 2001;288(3):666‐669. 11676494 10.1006/bbrc.2001.5824

[21] Thiry A , Dogne JM , Masereel B , Supuran CT . Targeting tumor‐associated carbonic anhydrase IX in cancer therapy. Trends Pharmacol Sci. 2006;27(11):566‐573. 16996620 10.1016/j.tips.2006.09.002

[22] Akurathi V , Dubois L , Celen S , et al. Development and biological evaluation of ^99m^Tc‐sulfonamide derivatives for in vivo visualization of CA IX as surrogate tumor hypoxia markers. Eur J Med Chem. 2014;71:374‐384. 24378650 10.1016/j.ejmech.2013.10.027

[23] Carloni Filho J , Santini AO , Nasser ALM , et al. Potentiometric determination of saccharin in commercial artificial sweeteners using a silver electrode. Food Chem. 2003;83:297‐301.

[24] Spencer M , Gupta A , Van Dam L , et al. Artificial sweeteners: a systematic review and primer for gastroenterologists. J Neurogastroenterol Motil. 2016;22(2):168‐180. 26932837 10.5056/jnm15206 PMC4819855

[25] Guillem‐Llobat X . Defining, regulating and using saccharin at the outset of the industrial food era (1888–1914). Appetite. 2012;59(3):905‐911. 22967818 10.1016/j.appet.2012.09.003

